# Pandemics Throughout the History

**DOI:** 10.7759/cureus.18136

**Published:** 2021-09-20

**Authors:** Shrikanth Sampath, Anwar Khedr, Shahraz Qamar, Aysun Tekin, Romil Singh, Ronya Green, Rahul Kashyap

**Affiliations:** 1 Cardiovascular Medicine, Mayo Clinic, Rochester, USA; 2 Division of Critical Care Medicine, Mayo Clinic, Mankato, USA; 3 Internal Medicine, Tanta University Faculty of Medicine, Tanta, EGY; 4 Post-Baccalaureate Research Education Program, Mayo Clinic, Rochester, USA; 5 Anesthesia Clinical Research Unit, Mayo Clinic, Rochester, USA; 6 Neurology, Allegheny Health Network, Pittsburgh, USA; 7 Family Medicine, Southern Hills Medical Center, TriStar Division, Hospital Corporation of America (HCA) Healthcare, Nashville, USA

**Keywords:** pandemics, cholera, influenza pandemics, plague, sars, ebola virus

## Abstract

As we move amidst the coronavirus disease 2019 (COVID-19) pandemic, we have witnessed tremendous distress, death, and turmoil of everyday life for more than one year now. However, they are not modern phenomena; deadly pandemics have happened throughout recorded history. Pandemics such as the plague, Spanish Flu, HIV, and Ebola caused deaths, destruction of political regimes, as well as financial and psychosocial burdens. However, they sometimes resulted in scientific discoveries. Understanding the mechanism of the emergence of these pandemics is crucial to control any spreading pandemic and prevent the emergence of a potential new one. Public health agencies need to work on improving the countries’ pandemic preparedness to prevent any future pandemics. The review article aims to shed light on some of the deadliest pandemics throughout history, information of critical importance for clinicians and researchers.

## Introduction and background

In 1666, the term "pandemic" was first used to describe a continuously spreading disease in a country. The words epidemic and pandemic were used broadly and often alternatively in many social and medical contexts during the 17th and 18th centuries [1]. However, as the terminology has developed throughout time, new concepts have emerged. The terms endemic, outbreak, epidemic, and pandemic express how frequent and geographically extent a disease is now compared to previously. They are not solely used to describe infections; they are also used to describe non-infectious conditions such as cancer and hypertension. To differentiate between these terms, an endemic disease affects a population in the same area, which could be a town, a country, or even a continent. An outbreak is defined as an unanticipated increase in the number of people who present with a health problem or the emergence of cases in a new location. An epidemic is a disease outbreak that spreads across a larger geographical area than its anticipated endemicity. A pandemic is an epidemic that expands to more than one continent [2].

There are many reasons why pandemics have happened and have become more likely to occur. Climate change holds a significant impact on the transmission of zoonotic infections by influencing the environment of their vectors [3]. Increased land use due to expanding human population also changes the distribution of these vectors [4]. Increased animal-to-human contact over the years increased the risk of transmission of zoonotic infections to human beings [5]. Moreover, antimicrobial resistance is one of the leading causes that increase the potential of a future pandemic [6]. The potential use of microorganisms as a biological weapon also raises the probability of the emergence of a future pandemic [7]. In addition, healthcare workers' shortage and underprepared health systems affect the ability to contain the situation in case a pandemic arises [8].

On the one hand, pandemics throughout history have had significant health consequences and represented a threat to the existence of humankind. Rapidly spreading infections such as COVID-19 can overwhelm the healthcare system and lead to limited access to health services and increased mortality rates for both communicable and noncommunicable diseases. They also had significant social, economic, and political impacts [9]. On the other hand, the recent pandemics managed to increase pandemic preparedness and led to efforts to mitigate the effects of pandemics [10]. In this article, we review some of the major pandemics that hit humans throughout the centuries. We focus on how they presented, how long they lasted, extent and speed of spread, how they were transmitted, their mortality burden, and how they ended or whether they still represent a threat to humanity. We also discuss the impacts of these pandemics on society and behavioral science and briefly describe the main management options.

## Review

Brief history of pandemics

Athenian Plague

The symptoms of the Athenian Plague of 430 BC were rash, headache, and conjunctivitis, progressing to abdominal cramps and hemoptysis, eventually leading to death in 7-8 days in many cases. The exact count is not available, but it is estimated that the plague killed around 25% of Athenians and people in the surrounding areas. War overcrowding is believed to be one of the significant contributors. The range of reported symptoms is so wide that many infectious diseases could have caused this outbreak. The disease that caused this outbreak remains unknown.

Antonine Plague

The Antonine Plague occurred between 165 to 180 AD. Troops brought the disease from their eastern campaigns in what is currently Iraq, which eventually spread into the entire Roman empire up till western Germany. Marcus Aurelius, emperor at that time, is one of the 5 million estimated deaths during the pandemic [11]. The symptoms as described by Galen included rashes, hemorrhagic pustules, bloody diarrhea, fever, and sometimes hemoptysis. Littman and Cunha later concluded after research that it was probably smallpox [12]. Decimating 33% of the Roman Empire, the Antonine plague did not restrict to a geographical area as the empire had an extensive political network connected to many different regions over a wide area [11].

Justinian Plague

Called after the Byzantine emperor at that period, the plague of Justinian hit the Mediterranean in 541 AC [13]. Symptoms were those of infection caused by Yersinia pestis: fever, cough, and dyspnea in pneumonic plague and groin or axillary buboes in bubonic plague. Some victims experienced hallucinations which were followed by fever, fatigue. Sometimes sore throat or diarrhea were the initial symptoms. It was transmitted from infected rats to humans through flea bites, and human-human transmission also propagated the disease. The plague of Justinian is estimated to have killed 60 percent of the Mediterranean world [14]. DNA extracted from the dental pulp of remains from burial pits from that period confirmed that this pandemic was caused by Yersinia pestis.

Black Death

The Black Death, also known as the Plague, was a bubonic plague pandemic that occurred from 1346 to 1353 in Europe, Asia, and Africa. It is considered to be the most fatal pandemic recorded in human history with a death toll of around 200 million people. Some estimates suggest that it managed to kill as much as 60% of Europe’s population [15]. Physicians at that time attributed the disease to miasma: corruption of air. After decades of research by various individuals and organizations, the Indian Plague Research Commission established in 1905 found an association between the plague and dead rats around the human habitat, and that rat flea was the vector. There are three clinical types of plague - Bubonic plague, where the patient suffers from sudden onset high fever; septicemic plague, where the patient has overwhelming septicemia and gangrene of nose, ears, and extremities due to disseminated intravascular coagulation; and then the pneumonic plague, which spreads through aerosols and causes hemoptysis and death. On average, the patients died in 7-10 days once the disease reached its peak [16].

The Seven Cholera Pandemics

The first cholera pandemic, also known as Asiatic cholera, began in 1817. It started in India and extended to Nepal, Indonesia, China, Japan, the Middle East, and parts of Russia. It was followed by six other pandemics, which happened in the period between 1827 to 1923 and spread to different continents, including Europe and the United States. The first six pandemics were transmitted by the classical biotype of Vibrio cholerae serogroup O1 through contaminated water. The seventh cholera pandemic, which is still ongoing, began in 1961. It started in Indonesia and spread to more continents and countries than the previous six pandemics, then became endemic in several world regions. It is transmitted by El Tor biotype strain of Vibrio cholerae through contaminated water and is still causing outbreaks across the world, the last of which happened in Somalia in March 2021. It is approximated that the first six cholera pandemics claimed about 1 million lives. The seventh cholera pandemic is estimated to cause around 2.86 million cholera cases globally every year, and of them, approximately 95,000 die every year. About 1.3 billion people are currently at risk of infection from cholera [17-22].

Spanish Flu

This deadly influenza pandemic originated in Kansas and spread due to the movement of the troops. It demonstrated nurses’ importance in healthcare while there were no antivirals or cure to be administered [23]. An H1N1 influenza of avian origin caused the 1918 Spanish flu, and it had high mortality for young individuals, which was a unique feature for this pandemic owing to their strong cytokine storm response to the virus. Also, there is evidence that suggests that there was some protection provided to those above the age of 30 because of a similar variant that spread during the “Russian flu” pandemic in 1889. Interventions such as quarantine and personal hygiene were the mainstay of prevention as no vaccine was developed yet. The pandemic killed enough young people to drop the US average life expectancy by 12 years [24]. The mild disease was associated with upper respiratory tract symptoms like sore throat, cough, pharyngitis, fever, myalgia, and prostration. Epistaxis was also seen in both mild and severe cases. Severe illness was characterized by respiratory distress, cyanosis, and pulmonary edema. Bronchopneumonia and ARDS were seen in fatal cases [25].

Asian Flu

Initiating from China in the first quarter of 1957, the H2N2 influenza virus, which originated from an avian influenza strain, spread to the United States, England, and Scotland within months [26]. The antigenic drift which caused the generation of influenza A subtype H2N2 would later cause periodic epidemics [27]. As usual infections with the flu, the patients presented with fever, chills, or myalgia. By the time it was resolved in April 1958, it had claimed more than 1 million lives globally [28].

Hong Kong Flu

Due to another antigenic drift, the H3N2 influenza virus emerged, containing two genes from an avian influenza virus and N2 from the agent of the 1957 pandemic [29]. Although it caused a milder disease compared to the Asian Flu, the virus was highly contagious. In July 1968, it originated from Hong Kong and quickly disseminated to other parts of the globe, starting from the United States. The major factor that affected the swiftness of the spread was the veterans returning from the Vietnam War. The mortality rate, despite being lower overall, was higher among the younger population [30]. Overall, it caused approximately one to four million deaths worldwide [31].

HIV/AIDS

The human immunodeficiency virus (HIV) has managed to kill 36 million people and has a current prevalence of around 36 million worldwide [32]. Although controllable by continuous antiretroviral treatment, total HIV cure is very unlikely as the “Berlin patient” was cured of HIV after administration of intensive chemotherapy, total body radiation, and bone marrow transplantation from a 32CCR5 homozygous donor. It was deduced that the elimination of CCR5 expressing cells is one of the bases of a complete cure [33]. It will require an epidemiologic and global public health intervention to end the HIV pandemic. As of 2014, UNAIDS issued a 90-90-90 treatment target for HIV which means that 90% of infected should have their infection diagnosed, 90% of the diagnosed receive ART, and 90% of those getting treated should achieve viral suppression [34]. Although mostly asymptomatic, 40%-90% of those affected might present with an acute retroviral syndrome, which occurs in 2-6 weeks postexposure to HIV-1 and includes fever, weight loss, fatigue, sore throat, lymphadenopathy, myalgia, nausea, headache, and diarrhea. Many other nonspecific findings/presentations can be seen, such as thrombocytopenia, leukopenia, aseptic meningitis, mononucleosis-like illness, and opportunistic infections [35].

Severe Acute Respiratory Syndrome

The severe acute respiratory syndrome coronavirus (SARS-CoV), which belongs to the Coronavirus genus in the Coronaviridae family, caused a worldwide epidemic that lasted from 2002 to 2003. SARS stands for severe acute respiratory syndrome which was the most common cause of death in this disease. This epidemic started in Guangdong province in China and extended to 29 countries in North America, South America, Europe, and Asia. Its intermediate hosts were bats, palm civets, and raccoon dogs. Its death toll was only 774 deaths. Seven months after its first appearance in November 2002, the SARS Pandemic was declared over in July 2003 due to remarkable global efforts to identify the virus, isolate cases, and contact tracing [21,22].

Swine Flu

Swine flu of 2009 affected 1/10th of the population swiftly, but it was not as deadly. However, it possessed the same character as Spanish flu, disproportionately killing the young due to the robust immune response. The number of people died was around 150000 to 25000. It was called “panicdemic” due to the alarm created by international health agencies, disproportionate to the effect of the pandemic [36]. Caused by the H1N1 Influenza virus, emerging in Mexico, swine flu mostly affected adolescents and young adults. The virus spread to 122 countries in 6 weeks due to global trade and travel and had three waves in spring, summer, and fall [37]. Patients suffered from high fever, cough, sore throat, myalgia, shortness of breath, and sometimes vomiting and diarrhea. The usual cause of death in severe cases was a respiratory failure [38].

Ebola

The Ebola virus disease (EVD) pandemic, which presents by hemorrhagic fever, emerged in Guinea in 2013. It affected 28000 and killed 11000 individuals. Many healthcare workers who volunteered to care for EVD patients also succumbed to this disease. The disease presents as an abrupt onset non-specific viral syndrome with symptoms such as high fever, myalgia, and fatigue. This was followed by vomiting and diarrhea, which led to dehydration and hypovolemia. In severe cases, the patient takes a turn for the worse and presents with gastrointestinal bleeding and mucosal hemorrhage. The cause of death usually was hypovolemic/hemorrhagic shock [39].

Coronavirus Disease 19

Caused by the severe acute respiratory syndrome coronavirus 2 (SARS-COV2), symptoms of coronavirus disease 19 (COVID-19) include fever, fatigue, productive cough, breathlessness, headache, anosmia, dysgeusia, and sore throat. Severe cases presented with complications such as ARDS, respiratory failure, cardiac failure, and septic shock [40-44]. The first case was reported to WHO from Wuhan on December 31, 2019, and it was declared as a global pandemic on March 11, 2020 [37,45,46-48]. The virus is thought to have originated from Wuhan province, China. Initially reported cases are considered to be from zoonotic source Huanan seafood market selling snakes, bats, and other wild animals [38]. Although the impact of different types of medications or conditions on the disease process is unknown, there is still not any effective treatment that is recommended by authorities [49,50]. Virus transmission is widely accepted to be through droplets expelled by sneezing, coughing, and talking. It can also be found in body fluids such as blood, semen, and ocular secretions. Findings suggest transmission through the air [51]. Globally, as of July 2021, there have been >190 million confirmed cases of COVID-19, including >4 million deaths, reported to WHO. As of July 2021, a total of >860 million people are administered with COVID-19 vaccine [52].

Table 1 summarizes the pandemics mentioned in this article [21,36,53].

**Table 1:** List of some of the pandemics that occurred throughout history.

Pandemic	Timeline	Area of emergence	Pathogen	Vector	Death toll
Athenian Plague	430-26 B.C.	Ethiopia	Unknown	Unknown	Unknown
Antonine Plague	165-180	Iraq	Variola virus	Humans	5 million
Justinian Plague	541-543	Egypt	Yersinia pestis	Rodents’ associated fleas	30-50 million
Black Death	1347-1351	Central Asia	Yersinia pestis	Rodents’ associated fleas	200 million
The Seven Cholera Pandemics	1817-present	India	Vibrio cholerae	Contaminated water	40 million
Spanish Flu	1918-1919	USA	Influenza A (H1N1)		50 million
Asian Flu	1957-1958	China	Influenza A (H2N2)		>1 million
Hong Kong Flu	1968	China	Influenza A (H3N2)		1-4 million
HIV/AIDS	1981-present	Central Africa	HIV		36 million
Severe acute respiratory syndrome coronavirus	2002-2003	China	Severe acute respiratory syndrome coronavirus	Bats	774
Swine Flu	2009-2010	Mexico	Influenza A (H1N1)		148000-249000
Ebola	2014-2016	Central Africa	Ebola virus	Unknown	11000
COVID-19	2019- July 2021 (ongoing)	China	SARS-Cov-2	Unknown	>4 million (ongoing)

Impacts of pandemics

Economic Impacts

Pandemics cause widespread significant morbidity and deaths in a brief period. It also leads to both short-term and long-term economic damage. An interesting contributor to the damage is the social aversion which the population develops due to the fear of pandemics. If a pandemic disproportionately affects the younger age group who are active contributors to the economy, it further increases the damage [10]. To avoid exposure to the virus, individuals reduce their interaction with suppliers, reducing the demand for goods and services that require close contact with persons [54]. Non-pharmaceutical interventions such as nationwide lockdowns, though necessary to stop the spread, affect the livelihoods of millions of people globally. According to the International Labor Organization, almost 400 million people lost their full-time jobs due to COVID-19 globally [55].

Societal Impacts

A century ago, pandemics were largely unexplained and uncontrolled, which wreaked havoc on society, economy, education, and health. This was mainly due to a lack of public health resources and knowledge about disease mechanisms [56]. Interestingly, the Black Death led to massive economic developments as society had to develop labor-saving and efficient technologies to compensate for the labor loss. However, at the same time, similar to COVID-19, the Black Death led to the promotion of unscientific treatments, shortage of doctors, and circulation of fake news. One possibility that arose from COVID-19, which could be beneficial, is an increase in work from home culture, which can incidentally accelerate the use of technology and increased productivity from the comforts of home [57]. The full impact of COVID-19 on the primary and secondary education system has not yet been fully revealed. Response to COVID-19 globally displayed that a consortium of scientists, healthcare workers, industries, and government policymakers is required for concerted management of the pandemic and that it cannot be solely managed at a national level [58].

Mental Health and Public Response Impacts

Books from the Middle Ages spoke about human behavior where the fear of contagion led men towards greed, corruption, and avarice, which paradoxically increased infection and led to mortality. Plagues have gotten enormous limelight in the world of literature [59]. During COVID-19, anxiety is high due to its new and relatively unknown nature compounded with lockdown isolation. The modern world is relatively unexposed to this feeling of sheer boredom and loneliness. Due to worldwide lockdown and ban on travels, individuals could not get vacations or weekends for relaxation and stress reduction. It was discovered through the study that 50% of the responders' mental health was average, and about 10% were languishing psychologically [60].

People cleared out shelves at supermarkets resulting in a shortage of food and essential supplies due to fear of COVID 19 lasting for a while and to regain control in their lives [61]. In the 20th century, massive advances were made like the discovery of antibiotics in the 1940s, the development of numerous vaccines, and improvements in disease surveillance strategies. Nevertheless, some things remain unchanged from the Black death till COVID-19, such as public disbelief of disease presence, disregard of government rules, propagation of myths and misinformation, unclear communication, and inadequate personal risk assessment. Suppression of news is still like the olden days where the government suppresses declaration of death tolls, and physicians are disrespected for warning the public of the arrival of a pandemic [56]. It was found that the younger population, people with comorbidities, and symptomatic patients were affected more psychologically [62].

Dilemmas

End-of-life-related decisions evoke intense feelings among doctors and nurses. Laurent et al, found in their study that during these times, the decisions of a nurse are influenced by their feelings towards the patient and those of a doctor by their feelings towards the patient’s family [63]. Caregivers’ struggles involve overcoming the imbalance between individual ethics and utilitarian ethics while making decisions. In such situations, healthcare workers had to make some imperfect but necessary decisions which lead to the disturbance of patient’s, kin’s, and the healthcare workers’ mental well-being. There is no single best solution during a pandemic. Societal ethics govern decision-making rather than individual ethics [64]. Pandemics shift the operative framework of clinicians from something considered “standard” to that of public health. What “I” ought to do changes to what “we” ought to do. Institutional obligations broaden from patients to now cover clinicians serving the patients [65]. Due to the absence of an appropriate framework for clinicians to navigate through moral dilemmas, confusion or harm may occur with lasting career implications [66].

Management

Quarantine

The chief physician of Ragusa built an establishment outside city limits for patients based on the contagion theory. He isolated the affected for 30 (trentine) days followed by an extension to 40 (quarantine) days. Thus the idea of quarantine was born [67]. Research shows that quarantined and suspect cases determine the trend of the epidemic. The peak of an epidemic is preceded by a static phase of quarantined and suspected cases [68]. A study in China showed that without the use of non-pharmacological interventions like quarantine during the COVID-19 outbreak, there would have been a 67-fold increase in incidence. In Europe, the same was found to be responsible for preventing over 3 million deaths in 11 countries [69].

Vaccination

China and India were the first to try active immunization by injecting variola into healthy individuals, which prevented scarring after a natural infection. This was followed by the works of Edward Jenner, Louis Pasteur, Calmette, and Geurin in the development of vaccines [70]. In the 1700s, Benjamin Jesty and Edward Jenner inferred that cowpox protects against smallpox as they observed that milkmaids did not develop scarring. As Jenner carried out an 18th-century clinical trial and informed the world of its success, inoculation quickly spread to the entire planet where people used poxvirus from arm lesions. Maurice Hilleman, widely regarded as one of the greatest microbiologists of all time, played a key role in advancing virology, epidemiology, and vaccine development. His contributions resulted in the creation of vaccines used for the prevention of many diseases including measles, mumps, chickenpox, meningitis, pneumonia, and hepatitis A and B. Vaccine evolution is based on some standard properties like knowing the etiology, developing systems for propagation, generating appropriate immune response and long-term immunity, clinical trials, choice of the type of vaccine and proper attenuation [71]. For more complex diseases like HIV/AIDS, for which we do not possess natural immunity, the development of vaccines needs more profound knowledge of the immune system [72]. Thirty-five years into the HIV pandemic, we have not managed to attain a successful preventive or therapeutic vaccine [73]. Several clinical trials aiming to produce a therapeutic HIV vaccine have successfully generated a robust immune response but without significant clinical benefits [74]. Table 2 shows some of the histories of vaccine development for the pandemics throughout history [70].

**Table 2:** History of vaccine development for some of the pandemics throughout the history.

Vaccine	Year developed	Type
Smallpox	1798	Live attenuated
Cholera	1896	Killed whole organism
Plague	1897	Killed whole organism
Yellow fever	1935	Live attenuated
Influenza	1942	Killed whole organism
Cholera	1993	Genetically engineered
Cholera	1994	Live attenuated
Influenza	2003	Cold adapted
Cholera	2009	Whole cell
COVID-19	2020	Messenger RNA (mRNA), Viral vector, and Protein subunit

Future directions

In the 21st century, there is a large amount of real-time data which describes the development of pandemics, achieving goals like, detection and monitoring of pandemic, assessment of the impact, analysis of the effectiveness of corrective measures, and planning appropriate control strategies [75]. Tele-ICU is an important tool during global health emergencies. By successfully implementing and deploying emerging technologies, the burden on healthcare would reduce as it would shift care from hospital to home or improvised hospital. This would enable timely intervention through detection of exacerbation or deterioration, rapid diagnosis, and treatment, etc [76]. Due to past and current inefficiencies in containing emerging infectious diseases, Onehealth, a transdisciplinary organization, has asked for a proactive approach to improving prevention and preparedness by improving coordination between different sectors. They intend to detect the virus at their source, where intervention can be applied before they spill over [77]. One Health is comprised of individuals from veterinary, environmental, and medicine fields [78]. Contact tracing has been in practice for years now but they have evolved to utilize mobile devices and wireless technologies [79]. Tripathi et al developed a wearable device called Easyband, which is a safety-aware mobility device. It enables the wearer to be made aware of infected individuals and hence avoid contact. Other features include - automatic listing of contact, Infection rate display of current location, and recommending self-isolation. It has the capability of working as a standalone device [80].

## Conclusions

Pandemics in the past had massive effects on the ethos of the human species. On the one hand, they have caused millions of deaths and had economic, societal, and mental health impacts. However, they led to significant scientific breakthroughs in terms of discovering the concept of quarantine, the development of vaccines, and novel treatment modalities. The COVID-19 pandemic has proved that we are still not prepared to detect, predict, or control the spread of novel pathogen pandemics. There is an unmet need to increase the awareness and exercise of pandemic preparedness to avoid the extreme burden on healthcare systems. It is also a stark reminder of the disparity between those who can afford health care and those who cannot afford it, especially in the countries without universal health care. National health policy organizations should prioritize widespread testing, contact tracing, quarantine, and vaccine development for crisis management of infectious disease pandemics. Future research needs to focus on developing better ways to prevent the emergence of newer pandemics and creating a uniform response.
